# Dose-dependent myopia-suppressing effect of 4-phenlybutyric acid eye drops in a mouse myopia model under masked condition

**DOI:** 10.1186/s12886-025-04213-6

**Published:** 2025-07-01

**Authors:** Shin-ichi Ikeda, Kohei Sone, Yoshihiro Takai, Takayuki Miyano, Kazuno Negishi, Kazuo Tsubota, Toshihide Kurihara

**Affiliations:** 1 Laboratory of Photobiology, Department of Ophthalmology, Keio University School of Medicine, 35 Shinanomachi, Shinjuku-ku, Tokyo, 160-8582 Japan; 2 Department of Ophthalmology, Keio University School of Medicine, 35 Shinanomachi, Shinjuku-ku, Tokyo, 160-8582 Japan; 3 Eye Care Product Development Division, ROHTO Pharmaceutical Co., Ltd, 6-5-4, Kunimidai, Kidugawa-shi, Kyoto, 619-0216 Japan; 4 Tsubota Laboratory, Inc, CRIK Shinanomachi E7, Bldg, 2, 9thFl. Keio University Shinanomachi Campus 35 Shinanomachi Shinjuku-ku, Tokyo, 160- 8582 Japan

**Keywords:** Myopia, Sclera, ER stress, 4-PBA

## Abstract

**Background:**

Myopia, characterized by excessive axial elongation of the eyeball, has become a significant global public health issue, particularly in Asia. Severe myopia increases the risk of ocular complications, including retinal degeneration and glaucoma. Recent studies have identified the role of scleral extracellular matrix remodeling and endoplasmic reticulum (ER) stress in myopia pathogenesis. Our previous study identified scleral ER stress as a critical factor in myopia development. This study aimed to investigate the therapeutic potential of 4-phenylbutyric acid (4-PBA), a chemical chaperone, in mitigating scleral ER stress and its effects on myopia progression in a mouse myopia model under masked condition.

**Methods:**

A lens-induced myopia (LIM) mouse model was utilized to evaluate the effects of 4-PBA administered as eye drops. Mice received varying concentrations of 4-PBA or phosphate-buffered saline (PBS) as a control. Changes in refractive error and axial length were measured to assess myopia progression. In addition, further experiments were also conducted under masked conditions, where the experimenter was unaware of the treatment was being applied, for the three conditions of 2%, 0.5% 4-PBA eye drops or vehicle treatment of LIM mice.

**Results:**

Administration of 4-PBA at concentrations of 0.5% and higher significantly suppressed the myopic shift in refraction and axial elongation associated with myopia compared to mice in the PBS control group. In a blinded, masked experiment, 2% and 0.5% 4-PBA eye drops also suppressed the progression of myopia in a dose-dependent manner.

**Conclusions:**

This study demonstrated that 4-PBA effectively mitigated myopia progression in a mouse model by targeting scleral ER stress. The dose-dependent suppression of myopic shifts and axial elongation under masked experimental condtion highlights the potential of 4-PBA as a therapeutic agent for managing myopia. These findings pave the way for further research and potential clinical applications in myopia treatment.

**Supplementary Information:**

The online version contains supplementary material available at 10.1186/s12886-025-04213-6.

## Background

Myopia, or nearsightedness, is a common visual disorder that has experienced a significant global increase in prevalence, particularly in Asian countries [1]. This condition, characterized by excessive elongation of the eyeball, represents a major public health concern due to its association with serious ocular complications such as retinal degeneration, glaucoma, and retinal detachment [2]. The development and progression of myopia are driven by complex and multifactorial mechanisms involving both genetic and environmental factors [3, 4].

Growing evidence has highlighted the critical role of the sclera, a dense fibrous connective tissue that defines the size and shape of the eye, in the pathogenesis of myopia [5–11]. Alterations in the extracellular matrix remodeling within the sclera contribute significantly to the excessive axial elongation observed in myopic eyes [7, 10, 12, 13]. The dynamic nature of the sclera and its ability to respond to various environmental and physiological cues make it a prime target for interventions aimed at controlling myopia progression.

Previous research showed that scleral endoplasmic reticulum (ER) stress is a fundamental event in the onset and development of myopia in mice [14]. Attenuating scleral ER stress through the administration of chemical chaperones, such as 4-phenylbutyric acid (4-PBA) or tauroursodeoxycholic acid, effectively suppressed scleral ER stress, myopia-associated scleral remodeling, and ultimately myopia development. Liquid chromatography-tandem mass spectrometry (LC-MS/MS) analysis confirmed the transfer of 4-PBA to the sclera following administration via eye drops. These findings suggest that targeting scleral ER stress with 4-PBA represents a promising approach for developing new myopia-inhibitory drugs.

Understanding dose dependency plays a pivotal role in developing new drugs, as highlighted by studies conducted by Bretz et al. and Viele et al., which emphasized the importance of characterizing dose-response relationships to ensure safety and efficacy [15, 16]. Although appropriate dose conversion methods between animal and human doses are required [17], animal models have been instrumental in advancing drug discovery and development [18]. These models enable early predictions of effective dosing to facilitate drug design [19].

This study aimed to evaluate the dose-dependent effects of 4-PBA eye drops on suppressing myopia using a mouse lens-induced myopia model under a blinded, prospective experiment.

## Methods

### Mice

Male C57BL/6J mice (3 weeks old; CLEA Japan, Yokohama, Japan) were housed in groups of five to six per cage under controlled environmental conditions, including a 12-hour light/dark cycle (lights switched at 8:00 AM/PM) and a temperature maintained at 23 ± 3 °C. Standard chow and water were provided ad libitum.

### Lens-induced myopia and 4-PBA or Atropine eye drop

Myopia induction in mice through negative lens wear, referred to as lens-induced myopia (LIM), was performed according to methods described in a previous study [20]. Briefly, mice were anesthetized using a combination of MMB, consisting of midazolam (4 mg/kg body weight; Sandoz K.K., Tokyo, Japan), medetomidine (0.75 mg/kg body weight; Orion Corporation, Espoo, Finland), and butorphanol tartrate (5 mg/kg body weight; Meiji Seika Pharma Co., Ltd., Tokyo, Japan). The anesthetic mixture was administered intraperitoneally at a volume of 0.01 mL/g body weight. Then, the skull was exposed and fixed with a special device for fitting frames over the eyes. A frame with a minus 30 diopter (D) lens was placed on the right eye, and a frame without a lens (as a control) was placed on the left eye.

Sodium 4-phenylbutyrate (Cayman Chemical, MI, USA, Catalog #: 11323) was dissolved in phosphate-buffered saline (PBS) at concentrations of 0.04%, 0.10%, 0.20%, 0.50%, 1.00%, and 2.00%. The prepared solutions were divided into 1.5 mL Eppendorf tubes and stored at -30 °C until use. Atropine sulfate monohydrate was purchased from FUJIFILM Wako Pure Chemical Ltd (Osaka, Japan), and dissolved in PBS at 1% concentration. During the LIM period, which spanned from 3 to 6 weeks of age (3 weeks), 4-PBA, 1% atropine or PBS (control) was administered as eye drops once daily (5 µL solution per eye). Axial length and refractive values were measured before and after the LIM period using spectral-domain optical coherence tomography (SD-OCT) (Envisu R4310, Leica Microsystems, Wetzlar, Germany) and an automated infrared photorefractor (Steinbeis Transfer Center, Tübingen, Germany). All measurements were performed under anesthesia with the MMB.

### Repeated ocular instillation toxicity study in pigmented rabbits

Pigmented rabbits (Kbl: Dutch, 14–17 weeks old for males, 13–16 weeks old for females: Kitayama Labes Co., Ltd., Nagano, Japan) were used in this study. Animals were treated with 2% 4-PBA or placebo control via ocular instillation 4 times daily at 2-hour intervals for 26 weeks. The dose volume for each instillation was 50 µL/eye. Six rabbits of each sex per group were assigned to the experiments. Body weights and food consumption were measured once in a week. A comprehensive ophthalmological evaluation was performed. Irritation reactions were examined 2 times during the dosing period in Weeks 4 and 24 using ophthalmoscope (Bx-α13; Neitz Instruments Co., Ltd., Tokyo, Japan) according to the McDonald-Shadduck grading method. IOP was examined twice during the dosing period in Weeks 10 and 23 using a tonometer (PNEUMATONOMETER MODEL 30 CLASSIC; Reichert, Inc., NY, USA). Each eye was measured 3 times, and the mean value was calculated as individual data. Electroretinography (ERG) was examined twice during the dosing period in Weeks 14 and 22. The animals were dark-adapted, the ERG was restored under anesthesia by isoflurane inhalation while the animal’s body temperature was maintained with a temperature controller (NS-TC10; Neuroscience Inc., WI, USA). The parameters were recorded in the order of standard combined rod-cone response and c wave.

### Blinded, masked testing of 4-PBA eye drops in LIM mice

The testing procedure involved three individuals with clearly defined roles. The first individual prepared the reagents (4-PBA or placebo) and labeled them as A or B for each experiment. The second individual assigned numbers to the mice (1 to 32 for each trial) and determined, through random assignment, which mice would receive eye drops labeled A or B. The third individual administered the eye drops and measured the axial length, following instructions provided by the second individual. Only the first individual was aware of the identity of A and B, ensuring that the second and third individuals remained blinded throughout the experiments.

**Fig. 1:**
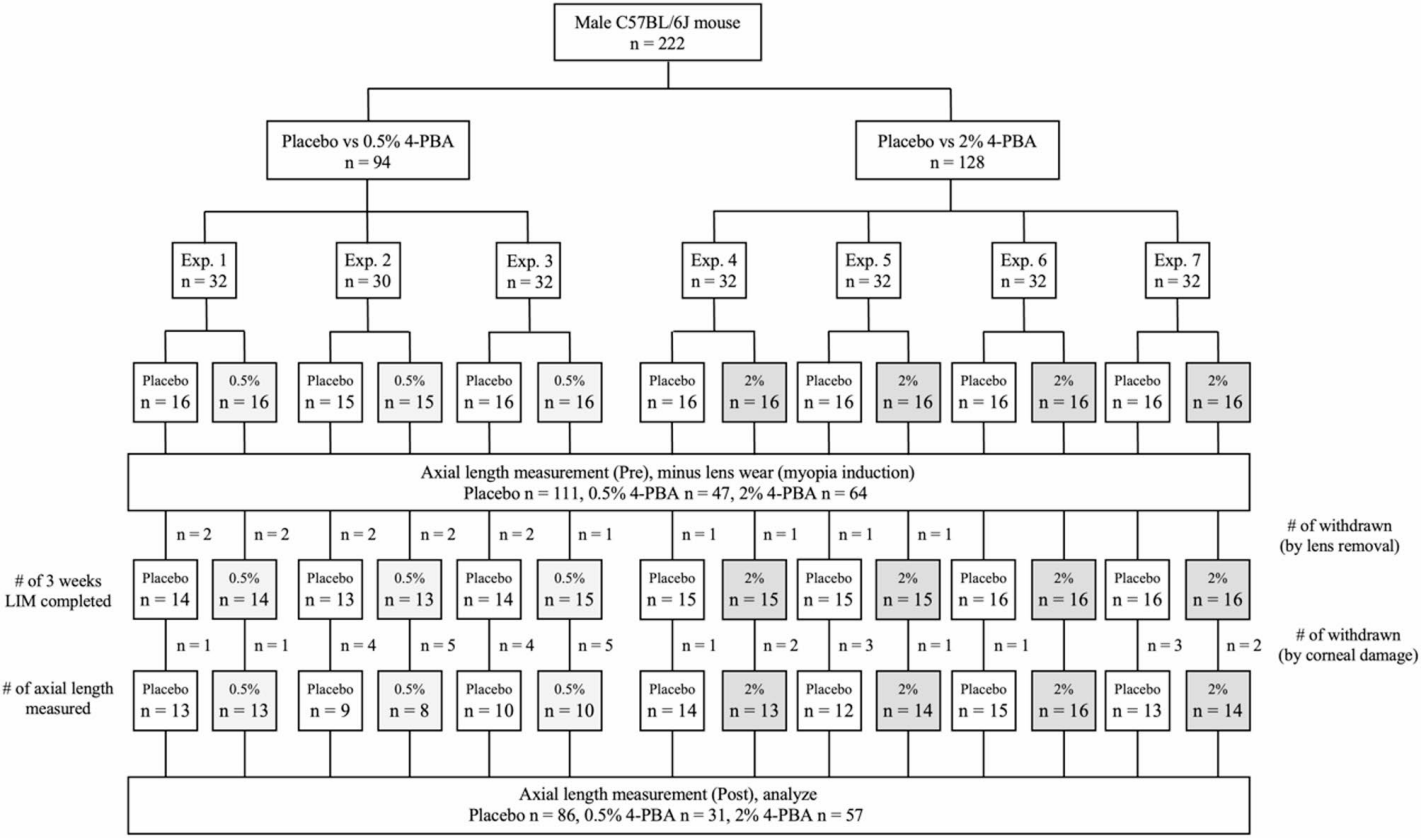
Flowchart of blinded, prospective study on 4-PBA eye drops in LIM mice

Seven independent trials were conducted. Four trials compared placebo with 2% 4-PBA, whereas three compared placebo with 0.5% 4-PBA. Each trial began with 32 mice (16 in each group) or 30 mice (15 in each group). Group allocation for each experiment was based on body weight at the time of pre-axial length measurement, recorded to the second decimal place, and performed using stratified random sampling in JMP 18 (JMP Statistical Discovery LLC, NC, USA). Exclusion criteria included cases where the negative lens detached during the LIM period or where OCT measurements were unfeasible due to corneal opacity or damage. Sample size calculations were informed by previous studies, accounting for approximately 40% of cases with lens detachment or unmeasurable conditions following LIM, to allow for comparisons across four groups. The combined data from all trials yielded final sample sizes of 85 mice in the placebo group, 31 in the 0.5% 4-PBA group, and 57 in the 2% 4-PBA group (Fig. 1). After the measurement, the animals used in those test were used in the following experiments or euthanized by cervical dislocation under anaesthesia with MMB.

### Western blotting

Following the above blinded, masked experiments, mice were euthanized by cervical dislocation under MMB anesthesia. Eyeballs were harvested from eight randomly selected mice from each of the placebo, 0.5%, and 2% 4-PBA groups. Sclerae were isolated and pooled in pairs to create one scleral sample for each analysis group (*n* = 4 per group for Western blotting). The isolated sclerae were homogenized in Pierce™ RIPA buffer (Thermo Fisher Scientific, MA, USA) containing Halt™ Protease and Phosphatase Inhibitor Cocktail (Thermo Fisher Scientific) using a Precellys Evolution homogenizer (Bertin Technologies, Montigny-le-Bretonneux, France). Protein concentrations in the homogenates were quantified using the BCA method and adjusted to 0.5 mg/mL with Laemmli sample buffer (Nacalai Tesque, Kyoto, Japan).

Scleral homogenates were separated on 10% polyacrylamide gels, transferred onto PVDF membranes (Merck Millipore, MA, USA), blocked with PVDF Blocking Reagent for Can Get Signal^R^ (TOYOBO, Kyoto, Japan), and incubated overnight with anti-ATF6 (1:3000, 73–505, Bio Academina, Osaka, Japan), phosphor-eIF2α (Ser51) (D9G8) XP Rabbit mAb (1:3000, #3398), eIF2α (D7D3) XP Rabbit mAb (1:5000, #5324), and b-actin (8H10D10) Mouse mAb (1:5000, #3700) (Cell Signaling Technologies Japan, Tokyo, Japan) at 4 °C. All primary antibodies were diluted with Can Get Signal™ Immunoreaction Enhancer Solution 1 (TOYOBO). After three washes with TBS-T, membranes were incubated with appropriate horseradish peroxidase-conjugated secondary antibodies (1:10000) and visualized using SuperSignal West Femto Maximum Substrate (Thermo Fisher Scientific).

### Statistical analysis

Differences between the control and experimental groups were compared using one-way or two-way analysis of variance, followed by either the least significant difference (LSD) post hoc or Tukey–Kramer tests to calculate statistical significance (GraphPad Prism software, version 8.0; GraphPad Software, San Diego, CA, USA). To confirm reproducibility, all experiments (excluding blinding tests) were conducted in at least two independent replicates.

## Results

### 4-PBA eye drops suppressed LIM-induced myopic shift in refraction in a dose-dependent manner

**Fig. 2:**
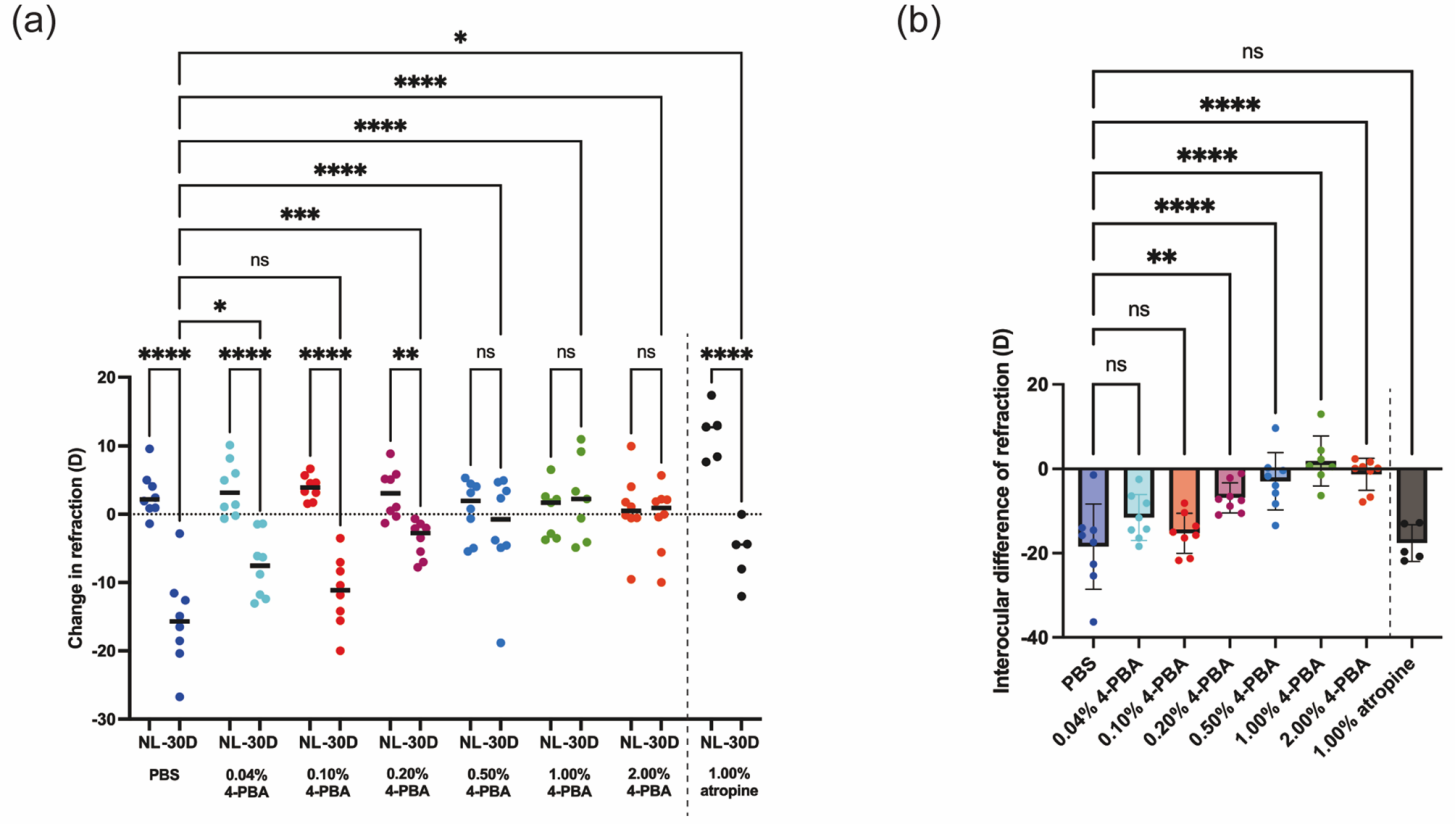
Dose-dependent effect of 4-PBA eye drops on myopic shift in LIM mice. (**a**) Changes in refraction over 3 weeks of LIM and administration of 4-PBA eye drops at different concentrations. Each group consisted of *n* = 8. *P*-values were determined using two-way ANOVA with Tukey multiple comparison test. *p < 0.05, **p < 0.01, ***p < 0.001, ****p < 0.0001. (**b**) Interocular differences in changes in refraction (calculated by subtracting the refractive change in the right, myopia-induced eye from the refractive change in the left, control eye). Each group consisted of *n* = 8 except 1.00% atropine group (*n* = 5). *P*-values were determined using one-way ANOVA with Dunnett multiple comparison test. **p < 0.01, ****p < 0.0001

First, we evaluated the effect of different concentrations of 4-PBA eye drop administration on LIM-dependent myopic shift in refraction in a mouse model. Figure 2a shows that PBS-treated control mice showed a myopic shift in refraction by wearing a -30 diopter lens (-30 D) compared to no lens (NL) eyes (NL eyes: 2.93 ± 3.34 D vs. -30 D eyes: -15.51 ± 7.02 D, *p* < 0.0001). In contrast, the 4-PBA eye drop groups showed no significant myopic shift in refraction with the − 30D lens at concentrations of 0.50% or higher (NL eyes: 0.84 ± 4.21 D vs. -30 D eyes: -2.09 ± 7.95 D in the 0.50% 4-PBA group, *p* = 0.2341; NL eyes: 0.41 ± 3.88 D vs. -30 D eyes: 2.31 ± 6.12 D in the 1.00% 4-PBA group, *p* = 0.4703; NL eyes: 0.76 ± 5.43 D vs. -30 D eyes: -0.52 ± 4.98 D in the 2.00% 4-PBA group, *p* = 0.6034). The intraocular difference in refraction became smaller in the group as the concentration of 4-PBA increased. At concentrations of 0.20% or more, the difference between the two eyes was significantly smaller than in the PBS-treated group (Fig. 2b).

### 4-PBA eye drops suppressed LIM-induced axial elongation in a dose-dependent manner

**Fig. 3:**
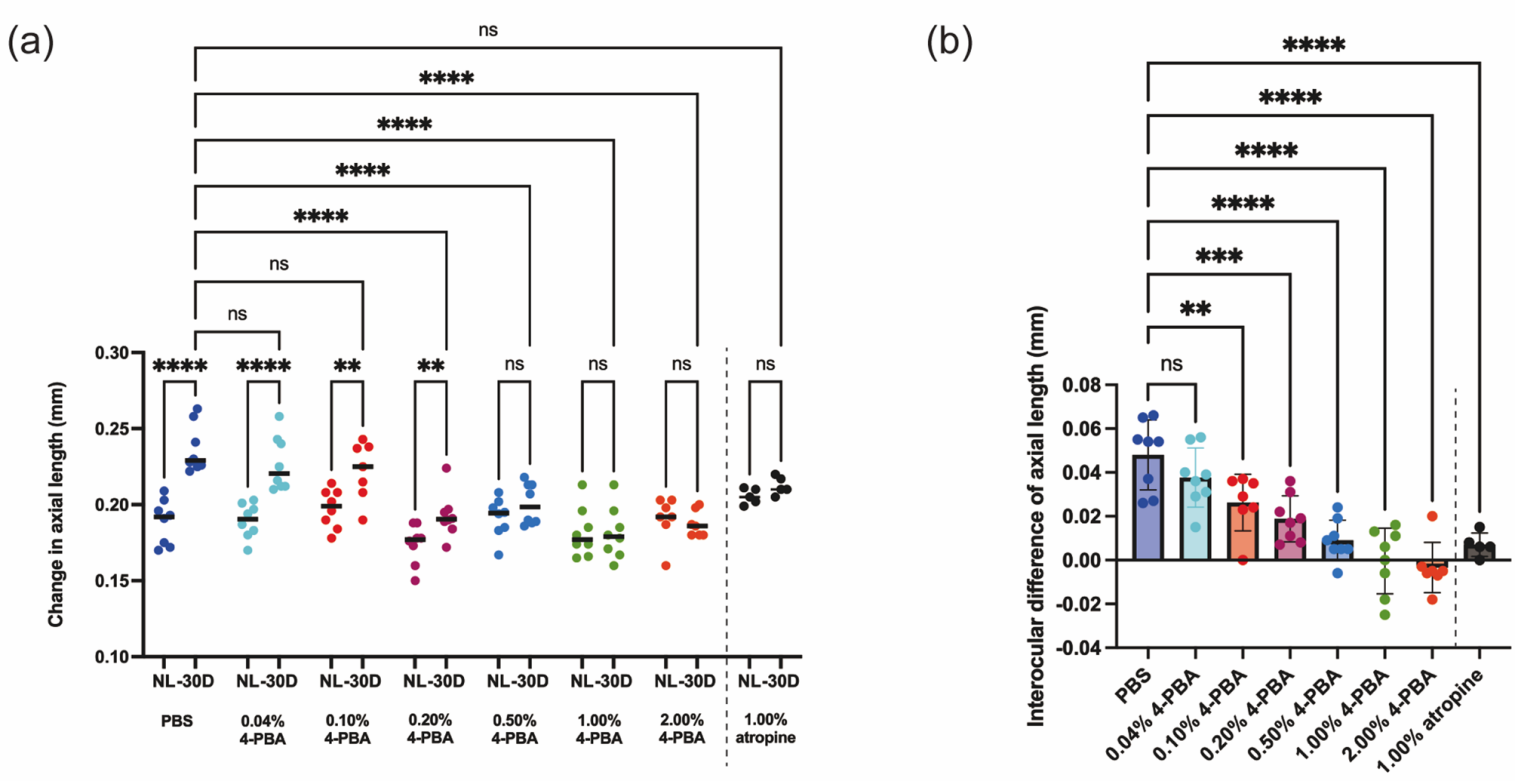
Dose-dependent effect of 4-PBA eye drops on axial elongation in LIM mice. (**a**) Changes in axial length over 3 weeks of LIM and administration of 4-PBA eye drops at different concentrations. Each group consisted of *n* = 8. *P*-values were determined using two-way ANOVA with Tukey multiple comparison test. *p < 0.05, **p < 0.01, ***p < 0.001, ****p < 0.0001. (**b**) Interocular differences in axial elongation (calculated by subtracting the axial elongation in the right, myopia-induced eye from the axial elongation in the left, control eye). Each group consisted of *n* = 8 except 1.00% atropine group (*n* = 5). *P*-values were determined using one-way ANOVA with Dunnett multiple comparison test. *p < 0.05, ***p < 0.001, ****p < 0.0001

The effects of different concentrations of 4-PBA eye drops on LIM-induced axial elongation were evaluated in a mouse model. PBS-treated control mice exhibited significant axial elongation when eyes were subjected to a -30 D lens compared to NL eyes (NL eyes: 0.189 ± 0.015 mm vs. -30 D eyes: 0.237 ± 0.016 mm, *p* < 0.0001; Fig. 3a), confirming the establishment of LIM. In contrast, 4-PBA-treated mice showed a reduction or elimination of axial elongation at concentrations of 0.5% or higher (NL eyes: 0.192 ± 0.013 mm vs. -30 D eyes: 0.201 ± 0.013 mm in the 0.50% 4-PBA group, *p* = 0.2237; NL eyes: 0.182 ± 0.016 mm vs. -30 D lens: 0.181 ± 0.017 mm in the 1.00% 4-PBA group, *p* = 0.9594; NL eyes: 0.191 ± 0.015 mm vs. -30 D eyes: 0.187 ± 0.008 mm in the 2.00% 4-PBA group, *p* = 0.6635). The intraocular difference in axial length decreased significantly in the 4-PBA-treated groups compared to the PBS group, with notable effects observed at concentrations of 0.20% or higher (Fig. 3b). Furthermore, since the NL eyes at each concentration were not fitted with minus lenses and received 4-PBA instillation, the effect on normal ocular growth can be evaluated by comparing the NL groups with each other. In this study, there were no significant differences among the NL groups at each concentration, suggesting that the concentrations of 4-PBA used in this study do not affect normal ocular growth.

### Comparing the myopia control effects of Atropine and 4-PBA Eyedrop

Atropine is currently the most extensively studied pharmacological agent for myopia control. Our comparative analysis of myopia-suppressing effects between 4-PBA and atropine revealed distinct efficacy profiles. While 1% atropine demonstrated suppression of axial elongation induced by minus lens wear (Fig. 3a and b), it showed no significant impact on refractive myopic shift (Fig. 2a and b). In contrast, 1% 4-PBA effectively inhibited both axial elongation and refractive changes (Figs. 2 and 3). These preclinical findings suggest that 4-PBA may possess superior myopia-controlling efficacy compared to atropine, at least in animal models.

### Safety evaluation of repeated administration of 2% 4-PBA in pigmented rabbits

To evaluate the safety of prolonged ocular administration of 2% 4-PBA, we conducted a 26-week study in pigmented rabbits. The animals received topical ocular instillation of either 2% 4-PBA or placebo four times daily. Parameters monitored included body weight, feed consumption, ocular irritation (Week 4 and 24), intraocular pressure (IOP; Week 10 and 23), and electroretinogram (ERG) measurements (Week 14 and 22). No statistically significant differences were observed between the placebo group and the 2% 4-PBA group across all evaluated metrics. These results suggest that repeated long-term ocular administration of 2% 4-PBA is well-tolerated and safe in this model (Supplemental Fig. 1a-d and Supplemental Tables 1 to 18).

### The myopia-inhibiting effect of 4-PBA was confirmed in a completely blinded test

To further validate the effect of 4-PBA eye drops in suppressing myopia development in the LIM mouse model, a prospective blinded test was conducted (Fig. 1). A total of 222 mice were randomly allocated to seven experiments, each comprising 32 mice, except for one experiment involving 30 mice. Three experiments tested placebo (*n* = 16 or 15) versus 0.5% 4-PBA (*n* = 16 or 15) test, whereas four experiments tested placebo (*n* = 16) versus 2% 4-PBA (*n* = 16) test. All mice underwent LIM and received a placebo or 4-PBA eye drops for 3 weeks. Mice were excluded if lenses detached during this period or if clear OCT images could not be obtained due to corneal or lens opacity at the 3-week post-measurement. The final group sizes included 86 mice in the placebo group (77.5%), 31 in the 0.5% 4-PBA group (65.9%), and 57 in the 2% 4-PBA group (89.1%).

**Fig. 4:**
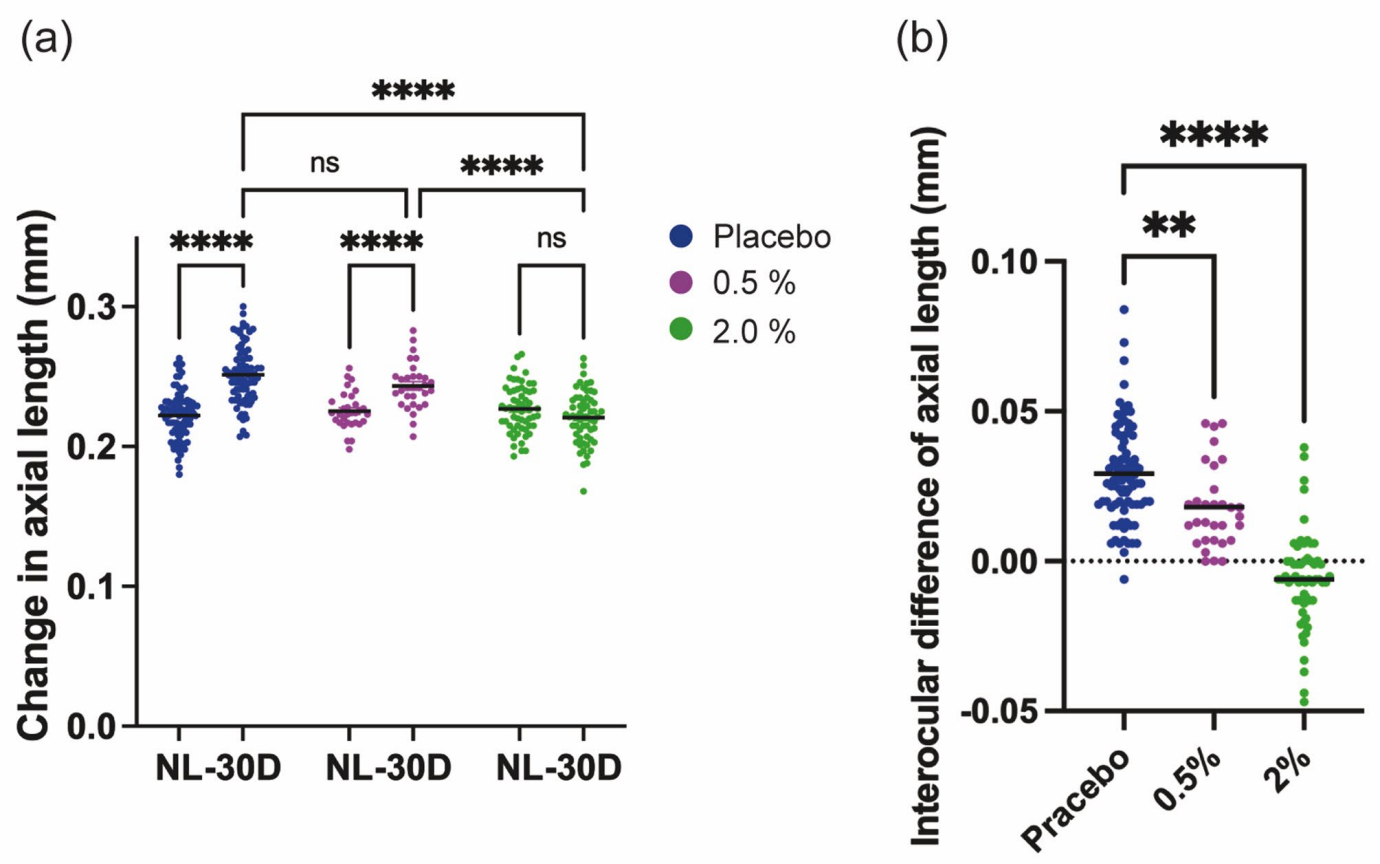
Verification of 4-PBA eye drops’ myopia-suppressing effect in a blinded animal study. (**a**) Changes in axial length over 3 weeks of LIM and administration of 0.5% or 2% 4-PBA (or vehicle) eye drops. Vehicle group: *n* = 85, 0.5% group: *n* = 31, 2% group: *n* = 59. *P*-values were determined using two-way ANOVA with Tukey multiple comparison test. ****p < 0.0001. (**b**) Interocular differences in axial elongation (calculated by subtracting the axial elongation in the right, myopia-induced eye from the axial elongation in the left, control eye). *P*-values were determined using one-way ANOVA with Dunnett multiple comparison test. **p < 0.01, ****p < 0.0001. LIM, lens-induced myopia; 4-PBA, 4-phenylbutyric acid; ANOVA, analysis of variance

In placebo-treated mice, -30 D eyes exhibited significant axial elongation compared to NL eyes (Fig. 4a.222 ± 0.017 mm vs. 0.252 ± 0.021 mm, *p* < 0.0001). The 0.5% or 2% 4-PBA groups showed reduced axial elongation (Fig. 4a.5%: 0.225 ± 0.013 mm vs. 0.243 ± 0.017 mm, *p* < 0.0001, 2%: 0.227 ± 0.017 mm vs. 0.221 ± 0.019 mm, *p* = 0.0628). These results were consistent with findings from the open-label studies (Fig. 3). As in the open-label study (Fig. 3b), the blinded test confirmed the dose-dependent myopia-suppressing effect of 4-PBA (Fig. 4b). These findings highlight 4-PBA eye drops as a promising therapeutic agent for myopia suppression.

### 4-PBA eye drops suppressed LIM-induced activation of unfolded-protein response in the sclera

**Fig. 5:**
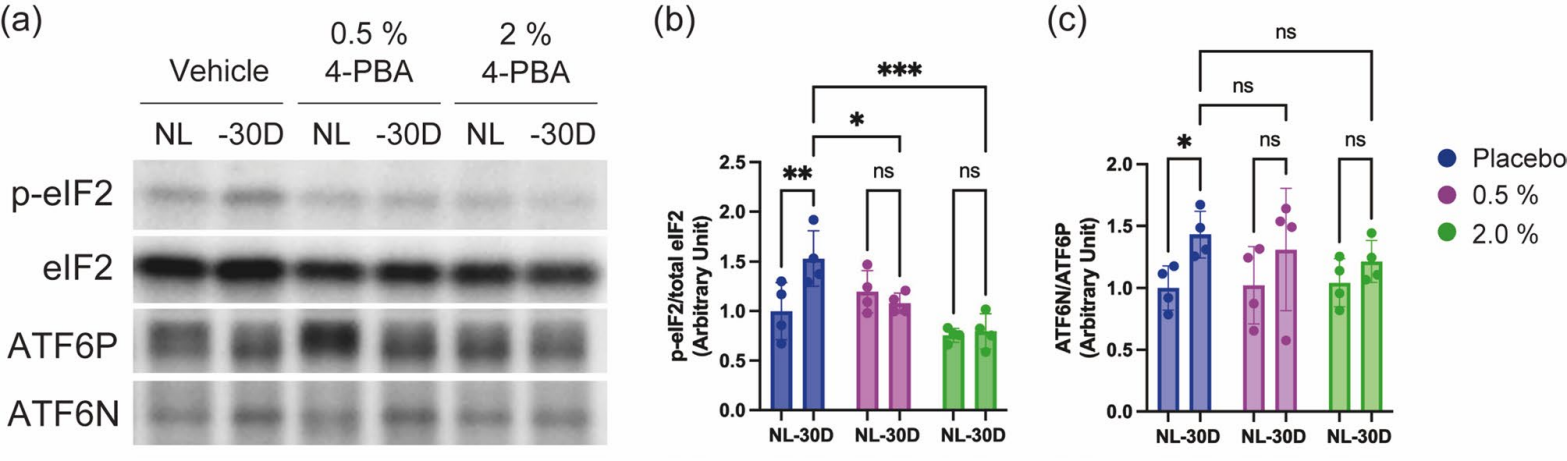
Verification of 4-PBA eye drops’ myopia-suppressing effect in a blinded animal study. (**a**) Representative blot images. (**b**) Quantified histogram depicting p-eIF2 normalized by total eIF2 protein. (**C**) Quantified histogram depicting ATF6N (active form) normalized by ATF6P (precursor form) protein. *P*-values were determined using two-way ANOVA with Tukey multiple comparison test. *p < 0.05, **p < 0.01, ***p < 0.001

ER stress triggers three unfolded protein response (UPR) pathways: inositol-response element 1 (IRE1)-X-box binding protein 1 (XBP1) signaling, PKR-like ER kinase (PERK)-eukaryotic translation initiation factor 2 (eIF2) pathway, and the activating transcription factor 6 (ATF6) pathway. Among these, PERK-eIF2 and ATF6 signaling are implicated in myopia development [14]. The effects of 0.5 and 2% 4-PBA on the activation levels of these pathways in the myopic sclera were evaluated. Activation of the PERK-eIF2 pathway was assessed through the ratio of phosphorylated eIF2 to total eIF2 expression, and ATF6 pathway activation was determined by the ratio of the cleaved form (ATF6N) to full-length ATF6 (ATF6P). In the vehicle-treated group, phosphorylation levels of eIF2 and ATF6N expression were higher in the − 30 D myopic sclera compared to the NL control sclera. Conversely, in the 2% 4-PBA-treated group, both eIF2 phosphorylation and ATF6N expression were comparable between NL sclera and − 30 D sclera (Fig. 5). In the 0.5% 4-PBA-treated group, eIF2 phosphorylation levels were similar in NL and − 30 D sclera (Fig. 5a and b), whereas ATF6N expression levels in the − 30 D sclera were slightly elevated compared to NL sclera, though the difference was not statistically significant (Fig. 5a and c).

## Discussion

In the development and research of a compound as a new drug, demonstrating dose dependency is crucial for determining the therapeutic index, optimizing dosing regimens, and understanding the direct link between dose-response relationships, pharmacokinetics, and pharmacodynamics [21, 22]. To facilitate the development of 4-PBA as a novel anti-myopia eye drop, we evaluated its concentration-response relationship in suppressing myopia using the LIM mouse model. The findings from both open-label studies and prospective blind tests demonstrated a clear dose-dependent effect of 4-PBA in myopia suppression. Furthermore, we demonstrated the safety of 2% 4-PBA eye drops through a long-term administration (26 weeks) study in pigmented rabbits.

Scleral ER stress is recognized as a fundamental cause of myopia development [14, 23, 24]. The chemical chaperone, 4-PBA, suppresses scleral ER stress and LIM-induced myopia. Conversely, tunicamycin, an ER stress inducer, can induce scleral ER stress and axial elongation in both mice and chicks [14, 23]. Additionally, bisphenol A, a compound widely used in plastic and resin manufacturing and identified as a potential health risk in aquatic systems, wildlife, and humans [25], induces scleral ER stress and myopia in C57BL6J mice, an effect that 4-PBA instillation prevents [26]. As the surface area of the conjunctiva is 17 times larger than that of the cornea and its permeability can be up to 29 times greater [27], drug delivery through the conjunctiva-sclera pathway appears effective for certain compounds. High-performance liquid chromatography-mass spectrometry (HPLC-MS) analysis confirmed the presence of 4-PBA in the sclera after eye drop administration [14], suggesting that 4-PBA reaches the sclera via this pathway and suppresses scleral ER stress. Notably, both 0.5% and 2% 4-PBA eye drops suppressed activation of the PERK and ATF6 pathways associated with myopia progression in LIM. Although ATF6 activation showed a non-significant trend, the overall findings underscore the potential of 4-PBA in mitigating scleral ER stress and myopia progression.

Atropine eye drops are currently the most widely recognized therapy for myopia control in human and animal models [28]. Their myopia-suppressing effect is thought to arise from their role as reversible antagonists of all five muscarine receptors 1–5 (MR1-5) and their ability to stimulate endogenous dopamine release, which inhibits the progression of form-deprivation myopia [29, 30]. However, the exact mechanisms remain elusive. Our findings demonstrated that 4-PBA eye drops, particularly at concentrations exceeding 1%, strongly suppress myopia progression by attenuating scleral ER stress, a mechanism distinct from that of atropine. In glaucoma treatment, combining drugs with different mechanisms of action has become a standard strategy [31]. Similarly, atropine is often used in combination with other optical treatments, such as orthokeratology, bifocal spectacle lenses, and multifocal soft contact lenses, to enhance its effectiveness in controlling myopia progression [32]. The combination of 4-PBA and atropine may offer improved control of myopia compared to atropine alone.

Atropine’s effectiveness may vary by ethnicity, with reduced efficacy observed in Caucasian populations. For instance, the WA-ATOM study, a 2-year randomized clinical trial (RCT) in Western Australia, found no significant difference in myopia progression between the 0.01% atropine group (-0.64 D) and the placebo group (-0.78 D) [33]. Similarly, an RCT in the United States reported no significant difference in spherical equivalent refraction (SER) at 24 months between the atropine (-0.82 D) and placebo (-0.80 D) groups [34]. Conversely, RCTs conducted in Asian countries, such as the ATOM2 and LAMP studies, demonstrated significant myopia suppression with low concentrations of atropine [35, 36]. Although the mechanisms underlying these racial differences remain unclear, 4-PBA, which suppresses myopia via a pathway distinct from atropine, could serve as a first-line option for individuals who do not respond well to atropine in myopia suppression. Importantly, in the present study, 1% atropine suppressed axial elongation but did not inhibit myopic shift in refraction consistent with previous mice study [20], and in such cases, combination therapy with 4-PBA may present a viable therapeutic option.

Blinded, masked testing, although a standard practice in clinical trials involving humans, is relatively uncommon in animal studies. According to one study, only 6.7% of 30 experiments employed blinding across all experimental stages, including assignment, intervention, implementation, outcome evaluation, and data analysis [37]. In a systematic review of 29 animal studies on FK506 (tacrolimus), only one study blinded researchers to the intervention and two blinded observers during outcome assessments [38]. The lack of blinding and other design flaws in animal studies has been cited as a significant factor contributing to the poor reproducibility of findings in human trials. To address these concerns, our study incorporated rigorous and fully blinded experimental protocols, with adequate replications and sample sizes, to validate the myopia-suppressing effects of 4-PBA. These well-controlled experiments further substantiated the findings of the open-label study and highlighted the potential of 4-PBA as a myopia inhibitor. Future clinical trials in humans are warranted to explore its efficacy.

4-PBA has been used in clinical trials for cystic fibrosis [39], sickle cell disease [40], and recurrent malignant glioma [41] and is approved for managing urea cycle disorders. Currently, no clinical trials have been conducted to investigate the use of 4-PBA for ocular diseases. However, ER stress represents a contributing factor in ocular conditions like cataracts, age-related macular degeneration, glaucoma, and diabetic retinopathy. Experimental studies have supported the cellular protective and therapeutic potential of 4-PBA in addressing these ocular diseases.

## Conclusions

In conclusion, this study demonstrated that the chemical chaperone 4-PBA eye drops inhibited myopia progression in a concentration-dependent manner by reducing scleral ER stress associated with myopia progression in an animal model. Furthermore, these findings, validated through a well-designed blinded study, provide critical data supporting the development of 4-PBA eye drops as a therapeutic agent to inhibit myopia progression.

## Electronic supplementary material

Below is the link to the electronic supplementary material.

Supplementary Material 1

Supplementary Material 2

Supplementary Material 3

## Data Availability

The datasets used and analysed during the current study are available from the corresponding author on reasonable request.

## Abbreviations

4-PBA: 4-phenylbutyric acid
LIM: Lens-induce myopia
PBS: Phosphate buffered saline
ER: Endoplasmic reticulum
LC-MS/MS: Liquid chromatography-tandem mass spectrometry
SD-OCT: Spectral-domain optical coherence tomography
NL: No lens
HPLC-MS: High-performance liquid chromatography-mass spectrometry
IRE1: Unfolded protein response
XBP1: X-box binding protein 1
PERK: PKR-like ER kinase
eIF2: Eukaryotic translation initiation factor 2
ATF6: Activating transcription factor 6
ATF6P: Activating transcription factor 6, precursor, full-length form
ATF6N: Activating transcription factor 6, nuclear, cleaved form
