# Supplementary figures and images for: Dose-dependent myopia-suppressing effect of 4-phenlybutyric acid eye drops in a mouse myopia model under masked condition
Source: BMC Ophthalmol. 2025 Jul 1;25:383. doi: 10.1186/s12886-025-04213-6 (PMC12220235; doi:10.1186/s12886-025-04213-6)

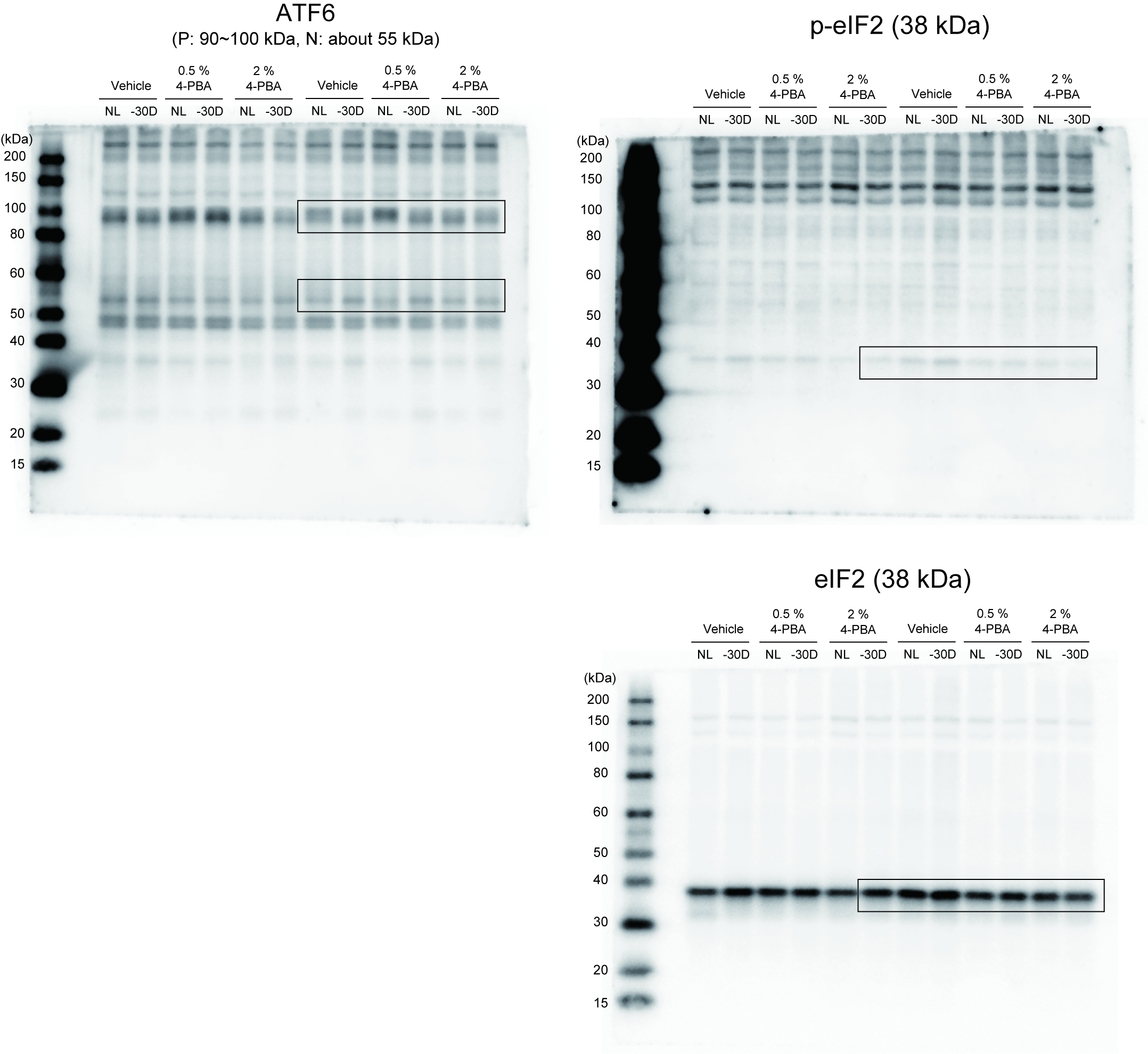

Supplement: Supplementary file 1 — Supplementary Material 1 [file 12886_2025_4213_MOESM1_ESM.tif]
