# Supplementary material for: Dose-dependent myopia-suppressing effect of 4-phenlybutyric acid eye drops in a mouse myopia model under masked condition
Source: BMC Ophthalmol. 2025 Jul 1;25:383. doi: 10.1186/s12886-025-04213-6 (PMC12220235; doi:10.1186/s12886-025-04213-6)
Supplement: Supplementary file 2 — Supplementary Material 2 [file 12886_2025_4213_MOESM2_ESM.pdf]

Supplemental Tabel 1 : Irritation reactions by McDonald-Shadduck method, Week 4, male

	Test Article Dose	Placebo												2 % 4-PBA											
Part of eyeball	Animal ID.	1001		1002		1003		1004		1005		1006		2001		2002		2003		2004		2005		2006	
	Side	R	L	R	L	R	L	R	L	R	L	R	L	R	L	R	L	R	L	R	L	R	L	R	L
Cornea a)	Score	0	0	0	0	0	0	0	0	0	0	0	0	0	0	0	0	0	0	0	0	0	0	0	0
Corneal cloudiness area b)		0	0	0	0	0	0	0	0	0	0	0	0	0	0	0	0	0	0	0	0	0	0	0	0
Corneal pannus c)		0	0	0	0	0	0	0	0	0	0	0	0	0	0	0	0	0	0	0	0	0	0	0	0
Fluorescein staining d)		0	0	0	0	0	0	0	0	0	0	0	0	0	0	0	0	0	0	0	0	0	0	0	0
Aqueous flare e)		0	0	0	0	0	0	0	0	0	0	0	0	0	0	0	0	0	0	0	0	0	0	0	0
Iris f)		0	0	0	0	0	0	0	0	0	0	0	0	0	0	0	0	0	0	0	0	0	0	0	0
Conjunctival congestion g)		0	0	0	0	0	0	0	0	0	0	0	0	0	0	0	0	0	0	0	0	0	0	0	0
Conjunctival swelling h)		0	0	0	0	0	0	0	0	0	0	0	0	0	0	0	0	0	0	0	0	0	0	0	0
Conjunctival descharge i)		0	0	0	0	0	0	0	0	0	0	0	0	0	0	0	0	0	0	0	0	0	0	0	0

R: Right, L: Left

a) Score 0 : Normal cornea

b) Score 0 : Normal cornea with no area of cloudiness

c) Score 0 : No pannus

d) Score 0 : Absence of fluorescein staining

e) Score 0 : Absence of visible light beam light in the anterior chamber (no Tyndall effect)

f) Score 0 : Normal iris without any hyperemia of the iris vessels.

g) Score 0 : Normal

h) Score 0 : Normal

i) Score 0 : Normal

Supplemental Tabel 2 : Irritation reactions by McDonald-Shadduck method, Week 24, male

Part of eyeball	Test Article Dose	Placebo												2 % 4-PBA											
	Animal ID.	1001		1002		1003		1004		1005		1006		2001		2002		2003		2004		2005		2006	
	Side	R	L	R	L	R	L	R	L	R	L	R	L	R	L	R	L	R	L	R	L	R	L	R	L
Cornea a)	Score	0	0	0	0	0	0	0	0	0	0	0	0	0	0	0	0	0	0	0	0	0	0	0	0
Corneal cloudiness area b)		0	0	0	0	0	0	0	0	0	0	0	0	0	0	0	0	0	0	0	0	0	0	0	0
Corneal pannus c)		0	0	0	0	0	0	0	0	0	0	0	0	0	0	0	0	0	0	0	0	0	0	0	0
Fluorescein staining d)		0	0	0	0	0	0	0	0	0	0	0	0	0	0	0	0	0	0	0	0	0	0	0	0
Aqueous flare e)		0	0	0	0	0	0	0	0	0	0	0	0	0	0	0	0	0	0	0	0	0	0	0	0
Iris f)		0	0	0	0	0	0	0	0	0	0	0	0	0	0	0	0	0	0	0	0	0	0	0	0
Conjunctival congestion g)		0	0	0	0	0	0	0	0	0	0	0	0	0	0	0	0	0	0	0	0	0	0	0	0
Conjunctival swelling h)		0	0	0	0	0	0	0	0	0	0	0	0	0	0	0	0	0	0	0	0	0	0	0	0
Conjunctival descharge i)		0	0	0	0	0	0	0	0	0	0	0	0	0	0	0	0	0	0	0	0	0	0	0	0

R: Right, L: Left

a) Score 0 : Normal cornea

b) Score 0 : Normal cornea with no area of cloudiness

c) Score 0 : No pannus

d) Score 0 : Absence of fluorescein staining

e) Score 0 : Absence of visible light beam light in the anterior chamber (no Tyndall effect)

f) Score 0 : Normal iris without any hyperemia of the iris vessels.

g) Score 0 : Normal

h) Score 0 : Normal

i) Score 0 : Normal

Supplemental Tabel 3 : Irritation reactions by McDonald-Shadduck method, Week 4, female

	Test Article Dose	Placebo												2 % 4-PBA											
Part of eyeball	Animal ID.	1101		1102		1103		1104		1105		1106		2101		2102		2103		2104		2105		2106	
	Side	R	L	R	L	R	L	R	L	R	L	R	L	R	L	R	L	R	L	R	L	R	L	R	L
Cornea a)	Score	0	0	0	0	0	0	0	0	0	0	0	0	0	0	0	0	0	0	0	0	0	0	0	0
Corneal cloudiness area b)		0	0	0	0	0	0	0	0	0	0	0	0	0	0	0	0	0	0	0	0	0	0	0	0
Corneal pannus c)		0	0	0	0	0	0	0	0	0	0	0	0	0	0	0	0	0	0	0	0	0	0	0	0
Fluorescein staining d)		0	0	0	0	0	0	0	0	0	0	0	0	0	0	0	0	0	0	0	0	0	0	0	0
Aqueous flare e)		0	0	0	0	0	0	0	0	0	0	0	0	0	0	0	0	0	0	0	0	0	0	0	0
Iris f)		0	0	0	0	0	0	0	0	0	0	0	0	0	0	0	0	0	0	0	0	0	0	0	0
Conjunctival congestion g)		0	0	0	0	0	0	0	0	0	0	0	0	0	0	0	0	0	0	0	0	0	0	0	0
Conjunctival swelling h)		0	0	0	0	0	0	0	0	0	0	0	0	0	0	0	0	0	0	0	0	0	0	0	0
Conjunctival descharge i)		0	0	0	0	0	0	0	0	0	0	0	0	0	0	0	0	0	0	0	0	0	0	0	0

R: Right, L: Left

a) Score 0 : Normal cornea

b) Score 0 : Normal cornea with no area of cloudiness

c) Score 0 : No pannus

d) Score 0 : Absence of fluorescein staining

e) Score 0 : Absence of visible light beam light in the anterior chamber (no Tyndall effect)

f) Score 0 : Normal iris without any hyperemia of the iris vessels.

g) Score 0 : Normal

h) Score 0 : Normal

i) Score 0 : Normal

Supplemental Tabel 4 : Irritation reactions by McDonald-Shadduck method, Week 24, female

Part of eyeball	Test Article Dose	Placebo												2 % 4-PBA											
	Animal ID.	1101		1102		1103		1104		1105		1106		2101		2102		2103		2104		2105		2106	
	Side	R	L	R	L	R	L	R	L	R	L	R	L	R	L	R	L	R	L	R	L	R	L	R	L
Cornea a)	Score	0	0	0	0	0	0	0	0	0	0	0	0	0	0	0	0	0	0	0	0	0	0	0	0
Corneal cloudiness area b)		0	0	0	0	0	0	0	0	0	0	0	0	0	0	0	0	0	0	0	0	0	0	0	0
Corneal pannus c)		0	0	0	0	0	0	0	0	0	0	0	0	0	0	0	0	0	0	0	0	0	0	0	0
Fluorescein staining d)		0	0	0	0	0	0	0	0	0	0	0	0	0	0	0	0	0	0	0	0	0	0	0	0
Aqueous flare e)		0	0	0	0	0	0	0	0	0	0	0	0	0	0	0	0	0	0	0	0	0	0	0	0
Iris f)		0	0	0	0	0	0	0	0	0	0	0	0	0	0	0	0	0	0	0	0	0	0	0	0
Conjunctival congestion g)		0	0	0	0	0	0	0	0	0	0	0	0	0	0	0	0	0	0	0	0	0	0	0	0
Conjunctival swelling h)		0	0	0	0	0	0	0	0	0	0	0	0	0	0	0	0	0	0	0	0	0	0	0	0
Conjunctival descharge i)		0	0	0	0	0	0	0	0	0	0	0	0	0	0	0	0	0	0	0	0	0	0	0	0

R: Right, L: Left

a) Score 0 : Normal cornea

b) Score 0 : Normal cornea with no area of cloudiness

c) Score 0 : No pannus

d) Score 0 : Absence of fluorescein staining

e) Score 0 : Absence of visible light beam light in the anterior chamber (no Tyndall effect)

f) Score 0 : Normal iris without any hyperemia of the iris vessels.

g) Score 0 : Normal

h) Score 0 : Normal

i) Score 0 : Normal

Supplemental Table 5 : Intraocular pressure, male pigmented rabbits

		Right mmHg		Left mmHg	
	Animal ID.	Week 10	Week 23	Week 10	Week 23
Placebo	1001	21.0	23.0	22.0	23.7
	1002	19.2	19.5	19.7	19.7
	1003	20.7	18.7	21.2	19.8
	1004	21.3	19.3	21.5	19.0
	1005	19.2	16.5	18.8	17.5
	1006	18.3	19.7	18.7	19.7
	Mean	20.0	19.5	20.3	19.9
	S.D.	1.2	2.1	1.4	2.1
2 % 4-PBA	2001	20.8	16.5	22.2	17.8
	2002	19.3	20.5	20.0	21.5
	2003	21.0	19.3	21.3	20.3
	2004	18.7	18.7	18.8	18.7
	2005	20.2	18.0	21.7	18.7
	2006	17.3	16.7	17.5	18.0
	Mean	19.6	18.3	20.3	19.2
	S.D.	1.4	1.5	1.8	1.4

Not significantly different from placebo

Supplemental Table 6 : Intraocular pressure, female pigmented rabbits

		Right mmHg		Left mmHg	
	Animal ID.	Week 10	Week 23	Week 10	Week 23
Placebo	1001	20.2	19.7	20.5	21.0
	1002	20.8	22.8	20.7	21.5
	1003	20.2	21.3	20.8	20.5
	1004	20.5	20.2	19.7	20.8
	1005	22.7	20.7	22.2	21.0
	1006	17.8	16.0	19.2	17.3
	Mean	20.4	20.1	20.5	20.4
	S.D.	1.6	2.3	1.0	1.5
2 % 4-PBA	2001	21.5	21.0	21.7	20.0
	2002	23.0	19.7	21.8	20.5
	2003	17.0	21.7	18.3	23.2
	2004	17.3	20.7	17.0	20.2
	2005	22.2	22.7	22.5	22.5
	2006	19.5	19.7	19.8	20.7
	Mean	20.1	20.9	20.2	21.2
	S.D.	2.6	1.2	2.2	1.3

Not significantly different from placebo

Supplemental Table 7 : Electroratinography, standard combined rod-cone response, **male** pigmented rabbits

		Latency, a wave			
		Right msec		Left msec	
	Animal ID.	Week 14	Week 22	Week 14	Week 22
Placebo	1001	13.2	13.5	14.4	15.9
	1002	15.3	15.0	15.0	16.5
	1003	17.1	17.1	17.4	17.1
	1004	10.8	18.3	12.6	17.4
	1005	16.2	17.1	15.6	16.8
	1006	16.5	15.0	16.5	16.8
	Mean	14.9	16.0	15.3	16.8
	S.D.	2.4	1.8	1.7	0.5
2 % 4-PBA	2001	17.1	17.4	15.9	17.1
	2002	16.8	18.0	17.1	17.4
	2003	17.1	18.3	18.3	18.0
	2004	17.7	17.7	18.6	18.6
	2005	17.1	18.0	16.8	17.4
	2006	16.2	13.8	15.3	16.2
	Mean	17.0	17.2	17.0	17.5
	S.D.	0.5	1.7	1.3	0.8

Not significantly different from placebo

Supplemental Table 8 : Electroratinography, standard combined rod-cone response, **male** pigmented rabbits

		Latency, b wave			
		Right msec		Left msec	
	Animal ID.	Week 14	Week 22	Week 14	Week 22
Placebo	1001	42.0	34.8	40.5	36.0
	1002	36.0	39.9	34.8	40.5
	1003	42.6	39.9	42.3	39.9
	1004	72.8	72.6	71.1	69.9
	1005	39.0	38.4	39.3	38.7
	1006	37.5	38.4	38.7	38.4
	Mean	45.0	44.0	44.5	43.9
	S.D.	13.9	14.1	13.3	12.8
2 % 4-PBA	2001	40.2	40.2	39.9	40.5
	2002	37.5	63.3	39.0	61.2
	2003	56.4	47.4	57.3	47.7
	2004	36.6	61.5	38.1	61.8
	2005	40.5	59.4	39.6	61.2
	2006	39.6	34.5	40.8	39.0
	Mean	41.8	51.1	42.5	51.9
	S.D.	7.3	12.1	7.3	10.8

Not significantly different from placebo

Supplemental Table 9 : Electroratinography, standard combined rod-cone response, **male** pigmented rabbits

		Amplitude, a wave			
		Right		Left	
		μV		μV	
	Animal ID.	Week 14	Week 22	Week 14	Week 22
Placebo	1001	102.5	92.77	97.17	85.94
	1002	105.5	80.57	131.3	95.21
	1003	100.1	115.7	137.2	104.0
	1004	73.24	69.82	57.62	97.17
	1005	109.4	102.1	105.0	101.1
	1006	105.0	111.8	110.8	124.0
	Mean	99.3	95.5	106.5	101.2
	S.D.	13.1	17.9	28.5	12.7
2 % 4-PBA	2001	96.19	94.24	97.17	102.5
	2002	96.68	103.0	87.40	95.21
	2003	80.57	85.45	95.21	97.17
	2004	50.29	85.45	73.24	87.40
	2005	76.66	105.5	73.24	100.6
	2006	129.9	128.4	108.9	96.19
	Mean	88.4	100.3	89.2	96.5
	S.D.	26.5	16.1	14.1	5.3

Not significantly different from placebo

Supplemental Table 10 : Electroratinography, standard combined rod-cone response, **male** pigmented rabbits

		**Amplitude, a-b wave**			
		Right<br>µV		Left<br>µV	
	Animal ID.	Week 14	Week 22	Week 14	Week 22
Placebo	1001	322.3	317.4	329.1	299.8
	1002	272.5	376.5	261.2	377.0
	1003	388.2	392.6	481.4	354.5
	1004	357.4	268.1	292.0	431.6
	1005	374.5	415.5	350.6	450.7
	1006	420.9	375.0	414.6	445.8
	Mean	356.0	357.5	354.8	393.2
	S.D.	52.3	54.5	81.2	60.1
2 % 4-PBA	2001	380.4	397.0	310.5	385.7
	2002	350.6	347.2	309.1	320.8
	2003	298.8	283.7	314.9	300.8
	2004	263.2	340.8	280.8	333.5
	2005	303.2	299.3	282.2	293.5
	2006	446.3	394.5	353.0	377.0
	Mean	340.4	343.8	308.4	335.2
	S.D.	66.3	46.9	26.4	38.5

Not significantly different from placebo

Supplemental Table 11 : Electroratinography, standard combined rod-cone response, **male** pigmented rabbits

	Animal ID.	Latency, c wave			
		Right msec		Left msec	
		Week 14	Week 22	Week 14	Week 22
Placebo	1001	2200	2160	2080	2000
	1002	2110	2070	1960	2080
	1003	1840	2040	1840	2020
	1004	2040	2060	2060	2050
	1005	2000	1970	1960	1970
	1006	2150	2200	2160	2230
	Mean	2,057	2,083	2,010	2,058
	S.D.	128	84	113	92
2 % 4-PBA	2001	1900	1860	1850	1870
	2002	1830	1790	1850	1820
	2003	2180	2020	2170	2020
	2004	1990	2090	2020	2100
	2005	1680	2000	1700	1990
	2006	1960	1950	2020	1960
	Mean	1923	1952	1935	1960
	S.D.	167	110	167	102

Not significantly different from placebo

Supplemental Table 12 : Electroratinography, standard combined rod-cone response, **male** pigmented rabbits

		Amplitude, c wave			
		Right		Left	
		μV		μV	
	Animal ID.	Week 14	Week 22	Week 14	Week 22
Placebo	1001	554.2	568.8	575.0	430.9
	1002	405.9	509.0	444.3	462.6
	1003	465.1	467.5	505.4	432.1
	1004	474.9	474.9	501.7	607.9
	1005	603.0	589.6	633.5	676.3
	1006	455.3	611.6	449.2	612.8
	Mean	493.1	536.9	518.2	537.1
	S.D.	72.1	61.3	73.7	107.7
2 % 4-PBA	2001	307.6	499.3	411.4	452.9
	2002	645.8	770.3	548.1	675.0
	2003	557.9	481.0	439.5	535.9
	2004	681.2	755.6	728.8	688.5
	2005	588.4	655.5	598.1	549.3
	2006	875.2	827.6	866.7	828.9
	Mean	609.4	664.9	598.8	621.8
	S.D.	185.1	146.4	174.2	135.2

Not significantly different from placebo

Supplemental Table 13 : Electroratinography, standard combined rod-cone response, **female** pigmented rabbits

		Latency, a wave			
		Right		Left	
		msec		msec	
	Animal ID.	Week 14	Week 22	Week 14	Week 22
Placebo	1001	17.1	16.2	17.1	17.4
	1002	17.1	13.8	15.6	14.7
	1003	17.4	16.2	15.9	13.5
	1004	18.0	15.9	18.3	18.0
	1005	15.9	16.5	17.4	17.4
	1006	15.0	15.3	16.2	17.4
	Mean	16.8	15.7	16.8	16.4
	S.D.	1.1	1.0	1.0	1.8
2 % 4-PBA	2001	16.2	16.5	16.5	16.8
	2002	17.1	16.8	16.2	16.8
	2003	17.7	16.8	16.2	17.1
	2004	16.2	17.7	15.9	17.7
	2005	16.8	16.8	16.8	16.8
	2006	14.7	16.2	15.3	16.8
	Mean	16.5	16.8	16.2	17.0
	S.D.	1.0	0.5	0.5	0.4

Not significantly different from placebo

Supplemental Table 14 : Electroratinography, standard combined rod-cone response, **female** pigmented rabbits

		Latency, b wave			
		Right msec		Left msec	
	Animal ID.	Week 14	Week 22	Week 14	Week 22
Placebo	1001	41.1	59.1	39.9	59.7
	1002	41.7	63.6	41.7	67.5
	1003	72.0	39.6	73.2	39.3
	1004	41.7	42.0	42.6	42.0
	1005	40.8	60.6	40.2	60.3
	1006	34.8	39.3	35.7	39.3
	Mean	45.4	50.7	45.6	51.4
	S.D.	13.3	11.5	13.8	12.6
2 % 4-PBA	2001	37.8	37.2	37.5	37.5
	2002	39.9	40.8	39.9	40.8
	2003	43.5	71.7	39.9	71.4
	2004	38.4	40.2	38.4	39.3
	2005	41.1	58.2	40.8	60.9
	2006	40.8	39.3	41.1	38.7
	Mean	40.3	47.9	39.6	48.1
	S.D.	2.1	13.9	1.4	14.4

Not significantly different from placebo

Supplemental Table 15 : Electroratinography, standard combined rod-cone response, **female** pigmented rabbits

		Amplitude, a wave			
		Right μV		Left μV	
	Animal ID.	Week 14	Week 22	Week 14	Week 22
Placebo	1001	112.3	87.40	108.9	96.19
	1002	87.89	83.98	79.10	83.98
	1003	77.64	85.45	77.64	86.91
	1004	106.4	97.66	78.13	87.40
	1005	118.7	127.0	132.3	125.5
	1006	115.2	111.3	121.1	127.0
	Mean	103.0	98.8	99.5	101.2
	S.D.	16.5	17.2	24.4	19.9
2 % 4-PBA	2001	102.5	95.70	92.29	92.77
	2002	140.1	103.0	128.4	102.1
	2003	104.5	87.89	96.19	117.7
	2004	88.38	81.05	65.43	100.6
	2005	87.40	106.6	100.1	106.9
	2006	113.3	119.1	86.91	107.9
	Mean	106.0	98.9	94.9	104.7
	S.D.	19.4	13.7	20.4	8.4

Not significantly different from placebo

Supplemental Table 16 : Electroratinography, standard combined rod-cone response, **female** pigmented rabbits

	Animal ID.	Amplitude, a-b wave Right μV Week 14	Amplitude, a-b wave Right μV Week 22	Amplitude, a-b wave Left μV Week 14	Amplitude, a-b wave Left μV Week 22
Placebo	1001	366.2	359.4	324.2	306.6
	1002	351.1	349.6	320.8	359.9
	1003	382.3	350.1	356.0	367.2
	1004	377.4	295.9	319.8	255.9
	1005	383.8	475.1	456.5	453.1
	1006	398.9	382.3	408.2	422.9
	Mean	376.6	368.7	364.3	360.9
	S.D.	16.4	59.3	56.5	72.6
2 % 4-PBA	2001	342.8	338.9	301.3	320.8
	2002	395.0	319.3	330.1	362.3
	2003	409.2	367.7	322.8	422.9
	2004	293.9	309.1	231.0	350.6
	2005	386.2	402.8	410.2	424.3
	2006	344.2	408.7	319.8	358.9
	Mean	361.9	357.8	319.2	373.3
	S.D.	43.0	42.2	57.5	41.6

Not significantly different from placebo

Supplemental Table 17 : Electroratinography, standard combined rod-cone response, **female** pigmented rabbits

		Latency, c wave			
		Right msec		Left msec	
	Animal ID.	Week 14	Week 22	Week 14	Week 22
Placebo	1001	1710	1920	1740	1920
	1002	2010	2210	2020	2210
	1003	2160	2540	2160	2530
	1004	2070	2240	2060	2130
	1005	2090	2010	2090	2000
	1006	2080	2130	2070	2000
	Mean	2,020	2,175	2,023	2,132
	S.D.	159	216	146	221
2 % 4-PBA	2001	2010	2250	1980	2280
	2002	2150	2030	2170	2040
	2003	2010	2130	1990	2100
	2004	1980	1990	2020	1930
	2005	1950	2110	1940	2020
	2006	2000	1990	2000	1970
	Mean	2017	2083	2017	2057
	S.D.	69	101	80	124

Not significantly different from placebo

Supplemental Table 18 : Electroratinography, standard combined rod-cone response, **female** pigmented rabbits

	Animal ID.	Amplitude, c wave Right μV Week 14	Right μV Week 22	Left μV Week 14	Left μV Week 22
Placebo	1001	673.8	765.4	604.2	666.5
	1002	496.8	520.0	505.4	407.7
	1003	684.8	610.4	578.6	606.6
	1004	692.1	727.7	664.1	711.7
	1005	808.1	655.5	764.2	610.4
	1006	891.1	810.5	743.4	716.6
	Mean	707.8	681.6	643.3	619.9
	S.D.	134.2	107.4	99.8	114.2
2 % 4-PBA	2001	666.5	618.9	670.2	615.2
	2002	758.1	593.3	741.0	716.6
	2003	615.2	572.5	581.1	634.8
	2004	650.6	634.8	599.4	684.8
	2005	566.4	773.9	601.8	661.6
	2006	921.6	961.9	838.6	843.5
	Mean	696.4	692.6	672.0	692.8
	S.D.	127.3	149.8	101.0	82.1

Not significantly different from placebo
