# Supplementary material for: Dose-dependent myopia-suppressing effect of 4-phenlybutyric acid eye drops in a mouse myopia model under masked condition
Source: BMC Ophthalmol. 2025 Jul 1;25:383. doi: 10.1186/s12886-025-04213-6 (PMC12220235; doi:10.1186/s12886-025-04213-6)
Supplement: Supplementary file 3 — Supplementary Material 3 [file 12886_2025_4213_MOESM3_ESM.pdf]

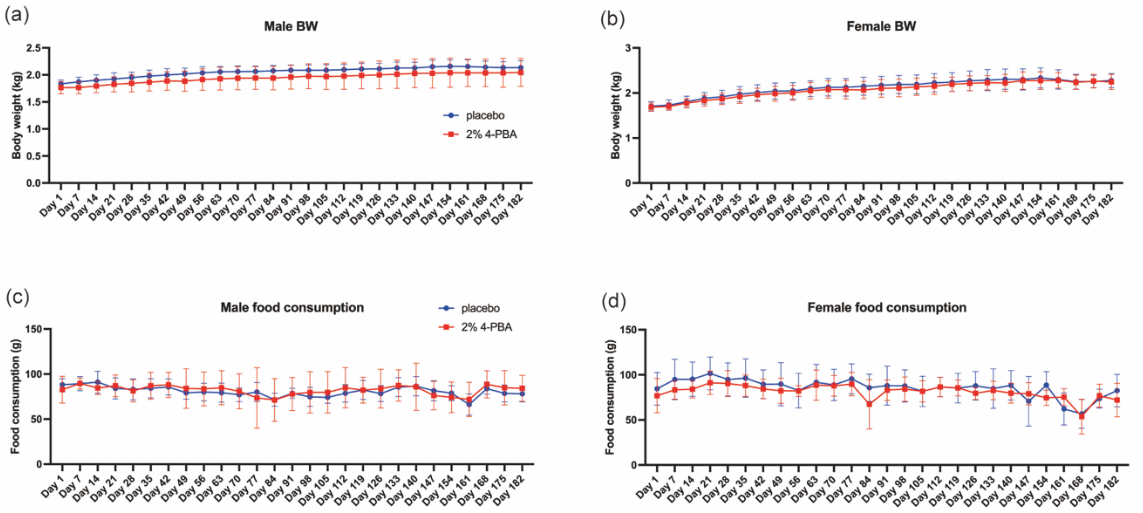

Supplemental Fig. 1 Effects of repeated instillation of 2 % 4-PBA over 26 weeks on body weight and food consumption.

(a) Body weight changes in male pigmented rabbits over 26 weeks. (b) Body weight changes in female pigmented rabbits over 26 weeks. (c) Food consumption in male pigmented rabbits over 26 weeks. (d) Food consumption in female pigmented rabbits over 26 weeks. No statistically significant differences were observed between the placebo and 2 % 4-PBA groups at any time point.
